# Recommendations for the effective use of T-cell–redirecting therapies: a Canadian consensus statement

**DOI:** 10.3389/fonc.2024.1446995

**Published:** 2024-11-26

**Authors:** Guido Lancman, Kevin Song, Darrell White, Tina Crosbie, Ismail Sharif, Marianne Emond, Muhammad Saleem Raza, Martine Elias, Rayan Kaedbey, Michael P. Chu

**Affiliations:** ^1^ Division of Medical Oncology and Hematology, Princess Margaret Cancer Centre, Toronto, ON, Canada; ^2^ The Leukemia/Bone Marrow Transplant Program of BC, BC Cancer Agency, Vancouver General Hospital, Vancouver, BC, Canada; ^3^ Queen Elizabeth II Health Sciences Centre and Dalhousie University, Halifax, NS, Canada; ^4^ Pharmacy Department, The Ottawa Hospital, Ottawa, ON, Canada; ^5^ Durham Region Cancer Centre, Oshawa, ON, Canada; ^6^ Pharmacy Department, Centre Hospitalier Universitaire de Québec-Université Laval, Quebec City, QC, Canada; ^7^ Dr. Everett Chalmers Regional Hospital, Fredericton, NB, Canada; ^8^ Myeloma Canada, Dorval, QC, Canada; ^9^ Segal Cancer Centre, Jewish General Hospital, McGill University, Montréal, QC, Canada; ^10^ Cross Cancer Institute, Edmonton, AB, Canada

**Keywords:** multiple myeloma, T-cell–redirecting therapy, bispecific antibodies, CAR T-cells, consensus statement, referral, adverse events

## Abstract

**Background:**

T-cell–redirecting therapies, such as bispecific antibodies and chimeric antigen receptor T-cells, exploit the cytotoxic capabilities of the immune system to destroy cells expressing specific surface antigens, including malignant cells. These therapies have demonstrated unprecedented rates, depth, and duration of responses in relapsed and refractory multiple myeloma. However, there are significant challenges in implementing these therapies into practice, which require multidisciplinary and multicenter coordination and significant healthcare resources to effectively manage these patients. So far, there are no Canadian guidelines for the effective implementation and use of T-cell–redirecting therapies.

**Methods:**

This consensus statement was developed based on three advisory meetings held in March, July, and November 2023. During these meetings, a panel of Canadian subject matter experts and representation from Myeloma Canada gathered to discuss the optimal procedures for the use of T-cell–redirecting therapies in the treatment of multiple myeloma. Members of the panel performed a thorough review of randomized clinical trials, real-world data, and other current literature, and provided their up-to-date clinical experience with T-cell–redirecting therapies in Canadian practice settings. Subsequently, asynchronous working groups were appointed to develop unified criteria for patient selection, appraise referral pathways, and devise strategies for management of short-term and long-term adverse events arising from the use of T-cell–redirecting therapies in multiple myeloma.

**Results:**

Here, we present recommendations for optimizing patient selection, referral pathways, and adverse event management in the Canadian practice setting. These recommendations are relevant for hematologists/oncologists, oncology nurses, pharmacists, nurse practitioners, physician assistants, and other providers who treat patients with multiple myeloma, as well as individuals with multiple myeloma and their care partners. These recommendations will be of interest to clinicians who treat patients with MM at community clinics and hospitals and who may be interested in referring patients for T-cell–redirecting therapy.

## Introduction

Multiple myeloma (MM) is a malignancy of plasma cells that most commonly affects older adults (1). Over 15,000 Canadians currently live with MM (2). According to the 2023 Canadian cancer statistics, approximately 3,900 individuals are diagnosed with MM and 1,700 individuals die from the disease each year (3). MM causes painful and distressing complications such as anemia, fractures, renal failure, infection, and weight loss (4). Over the past few years, there has been a rapid expansion of the therapeutic landscape, with a wide variety of novel therapies being developed and commercialized (5). These advances have been reflected in increases in overall survival (OS) over the past few decades (6). Emerging novel agents are providing patients with increasingly deep and durable responses and prolonged progression-free survival (PFS) in clinical trials (7–11).

The current paradigm for managing MM involves the use of triplet regimens that incorporate an anti-CD38 monoclonal antibody and dexamethasone along with a proteasome inhibitor (PI) and/or an immunomodulatory drug (IMiD) (12). Unfortunately, with each relapse, the available treatment options are reduced as the patient becomes refractory to more agents. According to a Canadian real-world study, the outcomes of triple-class exposed patients are poor, with a median OS of 12.6 months (95% confidence interval (CI) 10.7-16.2) (13). Ultimately, these challenges contribute to the general reduction in response rate and survival with each line of therapy, as noted by a recent real-world study (5). Treatment choice in the relapsed setting varies significantly between patients and jurisdictions, as clinicians must balance patient factors, treatment responses to prior lines of therapy (LOT), reimbursement considerations, tolerability of previous therapies, and potential future treatment options (14). This vast array of treatment options was convincingly demonstrated in the LocoMMotion study, in which 92 unique treatment regimens were identified among 248 triple-class exposed patients treated in a real-world setting (15). The results included a median PFS of 4.6 months (95% CI 3.9-5.6) and a median OS of 12.4 months (95% CI 10.3-not estimable) (15). Cytogenetic risk factors such as del(17p), t(4;14), and t(14;16) also contribute to worse outcomes, such as shorter duration of response and poor survival, in some patients (16, 17). Taken together, these data indicate a significant unmet need for more effective and tolerable therapies for relapsed or refractory MM (RRMM).

Recently, T-cell–redirecting (TCR) therapies have gained regulatory approval from Health Canada, including the chimeric antigen receptor (CAR) T-cell therapies ciltacabtagene autoleucel (cilta-cel) and idecabtagene vicleucel (ide-cel), and the bispecific antibodies (bsAbs) elranatamab, teclistamab, and talquetamab (18–22). These therapies are indicated for adults with MM who have received at least 3 prior LOT including a PI, an IMiD, and an anti-CD38 mAb, and who have demonstrated disease progression or are refractory to their last therapy (18–22). The provisional algorithm of care published by Canada’s Drug and Health Technology Agency (CADTH) outlines funding recommendations for RRMM therapy options, with recent updates to include cilta-cel (23).

Clinicians practicing in Canada would benefit from TCR therapy guidelines developed specifically for their practice settings. Several working groups, such as the American Society for Clinical Oncology (ASCO), the National Comprehensive Cancer Network (NCCN), the Society for the Immunotherapy of Cancer (SITC), and the International Myeloma Working Group (IMWG) have published clinical guidelines for the management of the adverse events (AEs) that are associated with TCR therapy and other immunotherapies (24–27). Nursing considerations and principles for patients receiving CAR T-cell therapy are also available (28, 29). The Canadian Myeloma Research Group (CMRG) has published recommendations for the management of MM complications, many of which apply to patients with MM who are treated with TCR agents, but these guidelines do not specifically address the safety of TCR therapy (30). BC Cancer has developed protocols for the use of teclistamab and for cytokine release syndrome (CRS) management (31, 32). However, few other guidelines regarding the use of TCR therapy in Canadian practice settings are available.

Health care practitioners who have not participated in clinical trials may lack hands-on experience with newly available agents. For example, in an American study, the majority (59%) of community health care providers reported barriers when caring for patients treated with the bsAb blinatumomab, and 86% of providers surveyed reported a need for guidelines and best practices (33). Care transitions, management of side effects, and a general lack of in-house expertise were among the challenges reported (33). The delivery of TCR therapies requires significant health care resources, collaboration between different health care centers, and multidisciplinary communication and organization. Canadian and international working groups and patient advocacy groups, including Myeloma Canada, the Association of Community Cancer Centers, ASCO, and the European Hematology Association/European Society for Blood and Marrow Transplantation (EHA/EBMT), have described a need for educational resources for both patients and clinicians to further optimize TCR therapy-based care (33–35).

Considering these challenges, Canadian health care professionals who treat patients with MM would benefit from expert guidance, based on recent clinical experience, on how to efficiently integrate TCR therapies into their practices and mitigate the risk of AEs. In this work, we describe best practices for the use of TCR agents within the Canadian health care context, including steps to be taken for selecting patients, referring patients, coordinating care between centers, and safely managing AEs. The focus of this paper is on the TCR therapies with the most mature supporting evidence and that are available, or soon to be available, in Canada. The recommendations provided here reflect our current experiences of managing patients receiving TCR therapies. It is expected that the recommendations will change in the future as new clinical data emerge, including evidence for the sequencing of therapies, and as best practices for adaptive adverse event mitigation and patient management are further refined.

## Overview of T-cell–redirecting (TCR) therapies

TCR therapies are among the most promising new and emerging agents for RRMM (36, 37). These agents utilize the cytotoxic properties of T-cells to destroy malignant plasma cells through the specific recognition of cell surface antigens. Two different classes of therapy have so far received regulatory approval: CAR T-cells and bsAbs (36, 37). CAR T-cell therapy is based on the *ex vivo* modification of autologous T-cells to direct their cytotoxic activity toward malignant plasma cells (38). BsAbs are engineered antibodies that are capable of recognizing two different antigens, creating an immunologic synapse between an immune effector cell and a malignant plasma cell (39).

Therapies directed against the B-cell maturation antigen (BCMA), a glycoprotein that is highly expressed on malignant plasma cells, have demonstrated significant clinical benefit (40). Other antigens that have been successfully employed include the G protein-coupled receptor, family C, group 5, member D (GPRC5D), a G protein-coupled receptor that is expressed on malignant plasma cells, and the Fc receptor homolog 5 (FcRH5), a differentiation antigen that is highly expressed on malignant plasma cells (41–44). The efficacy of these agents has been demonstrated in several clinical trials (recently published data are shown in **Table 1**).

**Table 1:** Efficacy data for TCR therapies.

Therapy	Pivotal trial and phase	Median follow-up	ORR (95% CI)^*^	CR or sCR (95% CI)	Median DOR (95% CI)	Median PFS (95% CI)	Median OS (95% CI)	Study population
CAR T-cell therapies								
**Ciltacabtagene autoleucel**	CARTITUDE-1 (phase 1b/2) (130)	33.4 mo (range 1.5-45.2)	97.9% (92.7-99.7) after 27.7 mo follow-up (72)	sCR 82.5% (73.4-89.4) after 27.7 mo follow-up (72)	33.9 mo (25.5-NE)	34.9 mo (25.2-NE)	NR	n=97; 88% triple-class refractory; 42% penta-refractory 13% EM disease, 24% high-risk cytogenetics 75% received bridging therapy
**Idecabtagene vicleucel**	KarMMa (phase 2) (165)	24.8 mo	73%	33%	10.9 mo	8.6 mo	24.8 mo	n=128; 26% penta-refractory; 84% triple refractory (11) 51% high tumor burden, 39% EM disease, 35% high-risk cytogenetics 88% received bridging therapy
Bispecific antibodies								
**Elranatamab**	MagnetisMM-3 (phase 2) (146)	15.9 mo	61.0% (51.8-69.6)	35.8%	NR	NR	NR	BCMA-naïve cohort: n=123; 96.7% triple-class refractory; 42.3% penta-refractory (9) 31.7% EM disease, 25.2% high-risk cytogenetics
**Talquetamab**	Monumen-TAL-1 (phase 2) (8, 81)	14.9 mo	74%	23%	10.2 mo (3.0-NR)	7.5 mo		0.4 mg/kg QW cohort: n=30; 77% triple-class refractory; 20% penta-refractory 37% EM disease; 22% high-risk cytogenetics
	MonumenTAL-1 (phase 2) (8, 81)	8.6 mo	73%	23%	7.8 mo (4.6-NR)	11.9 mo (61% censored) (81)		0.8 mg/kg Q2W cohort: n=44; 75% triple-class refractory; 20% penta-refractory 34% EM disease, 22% high-risk cytogenetics
**Teclistamab**	MajesTEC-1 (phase 1/2) (166)	1/2); 22 mo	63.0% (55.2-70.4) after 14.1 mo follow-up (7)	43%	24 mo (16.2-NE)	12.5 mo (8.8-17.2)	21.9 mo (16.0-NE)	n=165; 77.6% triple-class refractory; 70.3% penta-refractory (7) 17% EM disease; 25.7% high-risk cytogenetics

Data from the most recent publications reporting in-depth descriptions of efficacy for each agent (at the time of writing) are included. ^*^BCMA, B cell maturation antigen; CI, confidence interval; CR, complete response; DOR, duration of response; EM, extramedullary; mo, months; NE, not estimable; NR, not reached; ORR, overall response rate; OS, overall survival; PFS, progression-free survival; QW, every week; Q2W, every two weeks; sCR, stringent complete response.

Ciltacabtagene autoleucel (cilta-cel) and idecabtagene vicleucel (ide-cel) are second-generation, BCMA-directed CAR T-cell therapies that are Health Canada approved, but not yet integrated into clinical practice (18, 19, 45). These therapies have similar designs, but their chimeric antigen receptors have differences in the BCMA recognition domain: cilta-cel includes a single-chain variable fragment (scFv) with two camelid heavy chain domains that recognize distinct epitopes of BCMA, whereas ide-cel has a single mouse-derived variable fragment directed against a single BCMA epitope (46, 47). Within the past year, Health Canada issued regulatory approval of elranatamab and teclistamab, which are BCMA-directed bsAbs, and talquetamab, which is a GPRC5D-directed bsAb (20–22). None of the TCR therapies are currently reimbursed.

## Methods

This consensus statement was developed based on three advisory meetings held in March, July, and November 2023. The meetings included expert Canadian faculty from the fields of hematology and oncology who regularly treat patients with MM, as well as a representative of Myeloma Canada, who provided insights into patient experiences and concerns. Members of the panel performed a thorough review of randomized clinical trials, real-world data, and other current literature, and provided their up-to-date clinical experience with TCR in Canadian practice. Subsequently, asynchronous working groups were appointed to develop unified criteria for patient selection, appraise referral pathways, and devise strategies for the management of short-term and long-term AEs arising from the use of TCR therapies in MM.

## Results

### Rationale and recommendations for patient eligibility

The availability of healthcare resources, including healthcare professionals who are trained to administer TCR therapy and safely manage patients on therapy, has been a key challenge to the implementation of TCR therapy to date. In particular, the demand for CAR T-cell therapy will soon exceed supply (48). Although hospitalization is not necessarily required to administer TCR therapy, it may be required in response to AEs. To ensure widespread and equitable access to TCR therapies, identifying the patients who will benefit the most is a critically important step (49).

The indications of each therapeutic agent, as well as disease characteristics and other patient-specific factors such as performance status, comorbidities, and the nature of the patient’s support network, will all influence decision-making. The factors influencing patient selection described below should be viewed as flexible guidelines to be considered during a holistic decision-making process involving the clinician, patient, and care partner(s), rather than rigid criteria for eligibility. As more data become available, and as novel agents are used in earlier LOT and in combination, the eligibility criteria described here may be expanded.

#### Patient eligibility for CAR T-cell therapy

##### Minimum eligibility criteria for referral

Considerations for identifying the patients who are most likely to benefit from CAR T-cell therapy are arranged in descending order of importance in **Table 2**. Based on these criteria, patients who are eligible should be referred to a CAR-T center for consultation, where they may be further evaluated and a decision made on whether to start TCR therapy as their next step in treatment. Our panel encourages clinicians to consider the entire clinical picture of each patient, with more weight given to the factors such as prior treatment and performance status, and less weight given to social factors.

**Table 2:** Factors influencing patient selection for T-cell–redirecting therapies.

			Minimum criteria to qualify for referral	
	Category	Factor	Criteria for BCMA CAR T-cells	Criteria for BsAbs
**Most influence/weighting** 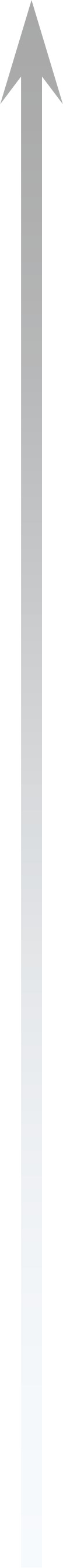	Treatment history	Prior lines of therapy	Follow the latest Health Canada indication; ≥3 prior lines of therapy^**^	
		Prior agents received	Follow the latest Health Canada indication (e.g., triple-class exposed, demonstrated progression on last therapy)	
	Performance status and organ function	ECOG performance status	ECOG 0-2	ECOG 0-3
		Active serious infection	Recommendation: control active serious infections (including hepatitis B and HIV) prior to therapy.	
		NYHA classification	I-II	I-III
		Creatinine clearance	≥30 ml/min Recommendation: <30 ml/min can be evaluated on a case-by-case basis	≥15 ml/min Recommendation: <15 ml/min can be evaluated on a case-by-case basis
		Pulmonary symptoms	Recommendation: if pulmonary disease is present, PFTs are recommended to evaluate patient.	
		Prior CNS comorbidities	Recommendation: patients with prior CNS comorbidities, including parkinsonism or a high risk of seizures (seizures within the prior 6 months) to be excluded. Patients with CNS MM should be considered with caution. For other comorbidities, review the latest indications and use clinical judgment.	Not relevant for bsAb therapy
**Least influence/weighting**	Social factors	Access to care	Within 30-minute transit of referral center for initial 30 days of treatment	Willing and able to travel for approximately the first month of treatment
		Care partner or support network	Access to a regular care partner/support network for 30 days post-treatment	After step-up dosing schedule, a care partner is not required if bsAbs are administered at a healthcare facility. If administered as outpatient, a care partner is beneficial for the first 7-14 days.

^*^BCMA, B cell maturation antigen; bsAb, bispecific antibody; CNS, central nervous system; ECOG, Eastern Cooperative Oncology Group; HIV, human immunodeficiency virus; NYHA, New York Heart Association; PFTs, pulmonary function tests.

^**^ Based on the Health Canada indications at the time of publication. For simplicity, we did not differentiate between bsAbs with different specificities. It is important to note that patient characteristics such as comorbidities and prior therapies may influence the choice between BCMA-directed bsAbs and GPRC5D-directed bsAbs.

According to the panel, the most important factor is prior treatment. At present, CAR T-cell therapy is indicated for patients who have received at least 3 prior LOT and who are refractory to their last LOT (50, 51). Since CAR T-cells are now being investigated in earlier LOT, which is likely to lead to changes in their indications, clinicians should follow the latest indications from Health Canada.

Next, patients’ overall fitness (performance status) and organ function should be considered. Patients should have an ECOG performance status of 0-2; patients with ECOG **≥**3 should be considered on an individual basis, with attention to the nature, etiology, and reversibility of the functional impairment. According to the expert panel, patients with ECOG **≥**3 are likely to have poor tolerance for CAR T-cell therapy. Age and frailty are important considerations, but age alone should not exclude patients from consideration. Age was not an eligibility criterion for CARTITUDE-1 or KarMMa, but the oldest patients in these studies were 78 years of age (10, 11). Real-world data indicate that some centers in the United States are willing to consider patients for CAR-T well into their eighties (52, 53). These data support the practice of looking beyond a patient’s chronological age when determining CAR-T eligibility.

Frailty may be a better assessment of a patient’s fitness for CAR-T beyond the ECOG performance status. Extensive work has been published in recent years on the impact of frailty on MM outcomes (54, 55). Although the impact of frailty on CAR-T treatment has yet to be fully explored, the available data show that frail patients may have lower response rates, PFS, and OS than non-frail patients; in one study, CAR-T efficacy was described as “reasonable” (56). However, frail patients and non-frail patients had similar rates of high-grade CRS and ICANS (56). Clinicians are encouraged to assess frailty either by applying their judgment and knowledge of their patients or by employing validated tools, during the work-up for CAR T-cell therapy. These assessments will be valuable to the referral center when determining treatment plans.

CAR T-cell therapy is associated with an increased risk of infection, and active acute infections should be treated prior to commencing lymphodepletion. Chronic infections such as hepatitis B and HIV should be well controlled before CAR-T treatment to minimize the risk of acute flares. Specifically, for patients with chronic hepatitis B, those with evidence of past infection (i.e., hepatitis B core antibody-positive but hepatitis B surface antigen-negative) should receive antiviral prophylaxis (e.g., entecavir or tenofovir). We recommend not offering CAR T-cell therapy to individuals who test positive for the hepatitis B surface antigen or for hepatitis B DNA, which would indicate a risk of viral reactivation (57). HIV that is well-controlled (e.g., negative viral load and CD4^+^ T-cell count **≥**200 cells/mm^3^) would not represent a contraindication to CAR T-cell therapy.

Many patients with MM have risk factors for cardiovascular disease (CVD) (58). CV toxicities, such as arrhythmias, cardiomyopathy, and venous-thrombolic events, have been noted in patients treated with anti-CD19 CAR-T, particularly in those who develop grade 3-4 CRS (59–62). The exact mechanism is not well understood, but some authors have postulated that this could occur through a similar mechanism as stress cardiomyopathy. The release of proinflammatory cytokines and the activation of prostaglandins during CRS are also thought to contribute (63). Although patients with MM receiving anti-BCMA CAR T-cell therapy may have a different CV risk profile than patients with leukemia or lymphoma requiring CAR T-cell therapy (e.g., prior anthracycline exposure), the risk of CV toxicity mediated by the physiological stress of CRS remains. Therefore, a careful evaluation of baseline cardiac function prior to CAR T-cell therapy is recommended. Clinical studies have set specific left ventricular ejection fraction (LVEF) parameters [e.g., ≥45% in CARTITUDE-1 (10)]. Our panel recommends a more pragmatic approach: patients planning to receive BCMA-directed CAR T-cell therapy should have at least moderate cardiac function as measured by minimal New York Heart Association (NYHA) heart failure symptoms (NYHA class I-II). However, even for patients with only NYHA class I or II symptoms, a cardiology consult should be considered if there is a history of arrhythmia.

Renal impairment is common among individuals with MM, occurring in 20-50% of patients at diagnosis (10, 11, 64). Although the pivotal trials CARTITUDE-1 and KarMMa excluded patients with severe renal disease (creatinine clearance (CrCl) <30 ml/min) (10, 11), the effectiveness and safety of CAR T-cell therapy are now being explored in this population (65–67). A study of patients with RRMM and impaired renal function who were treated with anti-BCMA CAR T-cell therapy found an improvement in renal function 6 months after treatment, especially among patients with light chain-type RRMM (65). Other work indicated that response rates to CAR T-cells among patients with mild or moderate renal impairment were similar to those of other patients with RRMM, and there were no differences in the rates of infection or CRS (66). A focused review noted that 18% of patients developed acute kidney injury after CAR T-cell therapy, but in most cases this was reversible (67). Therefore, a CrCl of >30 ml/min is desirable for eligibility for CAR T-cell therapy, and consideration may be given on a case-by-case basis for patients with CrCl <30 ml/min. Clinicians should also be aware that impaired renal function may influence the dosing of lymphodepleting therapy (67). Our panel recognizes that patient selection based on renal function is likely to evolve as more data emerge, and may be dependent on the specific TCR construct.

Pulmonary edema and dyspnea have been reported after CAR T-cell therapy, sometimes with a need for mechanical ventilation (68). Hypoxia may also occur as a manifestation of CRS (68). Pulmonary symptoms are not necessarily a contraindication, but patients with clinically-significant pulmonary disease should undergo pulmonary function tests (PFTs) to determine the extent of disease. CAR T-cell therapy may be contraindicated in patients with advanced pulmonary disease, including those who require home oxygen.

Patients with central nervous system (CNS) disease such as parkinsonism or a high risk of seizures (e.g., seizures within the past 6 months) are more likely to experience serious AEs and according to the expert panel, should not receive CAR T-cell therapy. Patients with CNS MM were excluded from the clinical trials of CAR T-cell therapy (10, 11) and should be considered with caution since they will be at high risk for neurotoxicity. Emerging data suggest that CAR T-cell therapy may be feasible in these patients (69–71). For instance, a retrospective analysis of 11 patients with CNS MM who were treated with CAR T-cell therapy reported an overall response rate (ORR) of 73% and CNS response rate of 100% after 3 months, with three patients experiencing ICANS and two patients experiencing delayed neurotoxicity (71). Larger studies with longer follow-up will be needed to confirm these results. For other CNS comorbidities, clinicians should review the latest Health Canada indication before coming to a decision to use CAR T-cell therapy based on clinical judgment and patient/care partner discussions. Baseline neurological assessment may be needed for a proper assessment of risk.

Finally, the patient’s social situation is an important factor in the success of therapy. Each patient will need access to an adequate support network before embarking on treatment with TCR therapies. Centers should work together with patients, their care partners and families, and other external support groups to establish robust care plans that will ensure 24-hour care partner support for at least 28 days after CAR T-cell therapy. The patient must also be willing to travel to a tertiary center for evaluation and be able to stay in proximity (within approximately 30 minutes’ drive) for the first 28 days after an infusion. Daily monitoring for the first week is required for ide-cel, whereas daily monitoring for the first two weeks and periodic monitoring for an additional two weeks are required for cilta-cel (50, 51).

##### Prognostic factors

Traditional risk factors, such as extramedullary disease (EMD), high-risk cytogenetics, and International Staging System (ISS) stage, remain important negative prognostic factors in patients treated with anti-BCMA CAR T-cell therapy. For example, in pivotal trials, patients with EMD had lower response rates and/or durations of response than the overall study populations (11, 72). We emphasize that patients with disease characteristics that are associated with a worse prognosis should not be excluded from TCR therapies. However, clinicians may wish to consider how these factors may impact the choice of treatment (e.g., CAR T-cells vs bsAbs) and treatment outcomes, especially for patients with “borderline” eligibility. Clinicians should be aware of the need for effective bridging therapy to avoid myeloma complications during the CAR T-cell manufacturing period (at present, about 6-8 weeks), especially for patients with high disease burden (73). Patients with rapidly progressing disease need readily available off-the-shelf therapies, and in these cases, bsAbs are likely to be more appropriate than CAR T-cells because of the lead time required for CAR T-cell manufacturing.

#### Patient eligibility for BsAb therapy

##### Minimum eligibility criteria for referral

Considerations for patient eligibility are arranged in descending order of importance in **Table 2**. Based on these criteria, patients who are eligible should be referred for bsAb therapy. As described above, clinicians should consider the entire clinical picture of each patient, with more weight given to the factors such as prior treatment and performance status, and less weight given to social factors. The bsAb eligibility criteria are more liberal than those for CAR T-cell therapy because lymphodepletion is not required and the severity of CRS and ICANS is lower (74, 75). The pathogenesis of CRS and ICANS is thought to involve proinflammatory cytokine signaling by activated macrophages, T-cells, and endothelial cells, although some aspects of pathophysiology vary between different TCR agents (74, 76). Severe CRS is less frequent with bsAbs than with CAR T-cells, which has been ascribed to the use of different mitigation strategies (e.g., steroid premedication and step-up dosing with bsAbs) as well as the subcutaneous administration of bsAbs and the larger population of T-cells resulting from CAR T-cell infusions (74).

At the time of writing, elranatamab and teclistamab are indicated for patients who have received at least 3 prior LOT, including a PI, IMiD, and anti-CD38 mAb, and who have progressed on their most recent therapy (77, 78). It is likely that bsAbs (as monotherapy or in combination) will move into earlier LOT in the future, and clinicians should follow the latest indications from Health Canada.

A clear picture of performance status must be provided to the referral center for all patients. The same information should be collected in advance for patients who will be treated in a community setting. Patients with an ECOG performance status of 0-3 may consider bsAb therapy. As a caveat, if the main comorbidities are due to disease symptoms, or if the reason for ECOG >3 is believed to be reversible, the patient is more likely to tolerate bsAb therapy and may be eligible. The clinician may also wish to consider the patient’s frailty status, with frail patients potentially needing more support to reduce the likelihood of treatment discontinuation. A systematic review of clinical trials demonstrated that although frailty is associated with worse outcomes, both frail and non-frail patients benefit from modern MM therapies (54). Furthermore, frailty often changes during the course of treatment (55).

BCMA-directed bsAb therapy is associated with grade 3-4 infections in up to 45% of patients (79). An analysis of MajesTEC-1 (at the time of writing, the bsAb trial with the longest follow-up; n=165) determined that after a median follow-up of 22 months, grade 3-4 infections had occurred in 52% of patients, including respiratory infections, COVID-19 and other viral infections, fungal infections, and GI infections (80). Fifteen individuals died from treatment-emergent infections during this trial, including 12 deaths from COVID-19 and 3 deaths from pneumonia (7). In contrast, talquetamab, a GPRC5D-directed bsAb, is associated with a much lower rate of infection, with grade 3-4 infections observed in 16-26% of patients, depending on the cohort (79, 81). Because of this risk, patients with uncontrolled infections (such as untreated hepatitis B or HIV) should not receive concurrent MM treatment with bsAbs. We recommend not offering bsAb therapy to individuals who test positive for the hepatitis B surface antigen or for hepatitis B DNA, because these indicate viral replication, which is linked to reactivation and the possibility of hepatic failure (57). However, individuals who are hepatitis B core antibody-positive (but surface antigen-negative) may receive prophylaxis (e.g., entecavir or tenofovir) and proceed with therapy. Well-controlled HIV (with a negative viral load and CD4^+^ T-cell count **≥**200 cells/mm^3^) is not a contraindication.

BsAb treatment can lead to damage to the CV and renal systems (82, 83). Proinflammatory cytokines released during CRS may lead to acute kidney injury, with factors such as fluid loss and infection also contributing (83, 84). Tumor lysis syndrome may result in hypocalcemia, hyperkalemia, and hyperuricemia, leading to cardiotoxicity and nephrotoxicity (84). According to an analysis of the US Food and Drug Administration’s Adverse Events Reporting System (FAERS), the most common CV AEs are bleeding, hypotension, thromboembolic disease, arrhythmias, and heart failure (85). These AEs represented 12.5% of the events reported to FAERS and occurred less frequently than non-CV events, but were associated with higher mortality (85). The study did not determine whether these events were treatment-related (85). In our panel’s clinical experience, CV events are rare, but we recommend that patients planning to receive BCMA-directed bsAb therapy have relatively good CV function as measured by mild to moderate NYHA heart failure symptoms (NYHA class I-III). Renal function should be adequate (i.e., CrCl ≥15 ml/min), but patients with lower values, including patients on hemodialysis, may be evaluated on an individual basis. In a series of seven patients with severely impaired renal function who were treated with teclistamab (86), response rates and safety were similar to those observed in MajesTEC-1, which required patients to have an estimated glomerular filtration rate (eGFR) ≥40 ml/min/1.73 m^2^ (7). No neurotoxicity or infection occurred in these patients (86). BsAbs are not excreted renally and cause minimal acute kidney injury (83).

For bsAbs that target BCMA (e.g., teclistamab, elranatamab), patients with clinically-significant pulmonary disease should undergo baseline PFTs to evaluate the severity of symptoms. This is because anti-BCMA bsAbs are associated with a risk of severe infection, including increased vulnerability to COVID-19 and other serious respiratory infections (87). Individuals with advanced pulmonary disease, including a requirement for home oxygen, may not be able to tolerate bsAb therapy.

In contrast to CAR T-cell therapies, CNS comorbidities are a lesser concern for bsAb therapy. BsAbs are unlikely to cross the blood-brain barrier, and immune effector cell–associated neurotoxicity syndrome (ICANS) is rarely severe (74). Neurotoxicity with bsAbs often occurs together with CRS and resolves after the CRS has been treated (88). Therefore, the risk of seizures is not as consequential as with CAR T-cells. Patients with stable epilepsy may be appropriate candidates.

Centers should work holistically with the patient, their care partners and family, and external groups (such as patient support groups) to ensure a strong support network. Patients living at a distance from the referral center should be willing to travel for approximately the first month of treatment; many community hospitals will soon be able to provide bsAb treatment starting at the second cycle. If the first cycle of bsAb therapy is administered on an outpatient basis, a care partner or other reliable support is required for the first 7-14 days (during step-up dosing).

##### Prognostic factors

High-risk cytogenetics and paraskeletal disease are associated with lower treatment response rates in MM, and this is also likely to apply to bsAb therapy. EMD was identified as a predictor of poor response for several bsAbs. For example, in the MonumenTAL-1 (n=30, n=44) and MajesTEC-1 (n=165) clinical trials, the ORRs to talquetamab were 40.0-45.5% among patients with EMD (n=11, n=15), and the ORR to teclistamab was 35.7% in the same population (n=28) (7, 8). The ORRs for the overall study population were 64-70% for talquetamab and 63.0% for teclistamab (7, 8). In MagnetisMM-3 (n=123), patients with EMD (n=39) had an ORR to elranatamab of 38.5% vs the ORR of 71.4% in patients without EMD (9). These disease characteristics should not prevent patients from receiving bsAbs, but may be considered as part of a holistic evaluation of the patient and shared decision-making. To increase the likelihood of response, patients should be referred for consideration of bsAb therapy at the earliest sign of relapse on their prior therapy. Patients with EMD may be considered for alternative treatments in clinical trials [e.g., cereblon E3 ligase modulators (CELMoDs) (89, 90) or novel combination therapies (91–93)] or tumor debulking [with dexamethasone, cisplatin, doxorubicin, cyclophosphamide and etoposide (DPACE) or a similar regimen (94)] prior to bsAb therapy. Clinicians may wish to consider bsAbs rather than CAR T-cells for patients with rapidly progressing disease because bsAbs do not require lead time for the manufacturing of individualized products. For other patients, the decision between bsAbs and CAR T-cell therapy will be complicated, and clinicians should engage with patients and their care partners to reach a shared decision. The factors in **Table 2** should be considered along with patient and care partner preferences, financial considerations, local resources, and access to different TCR agents.

### Referral pathway

**Figure 1:**
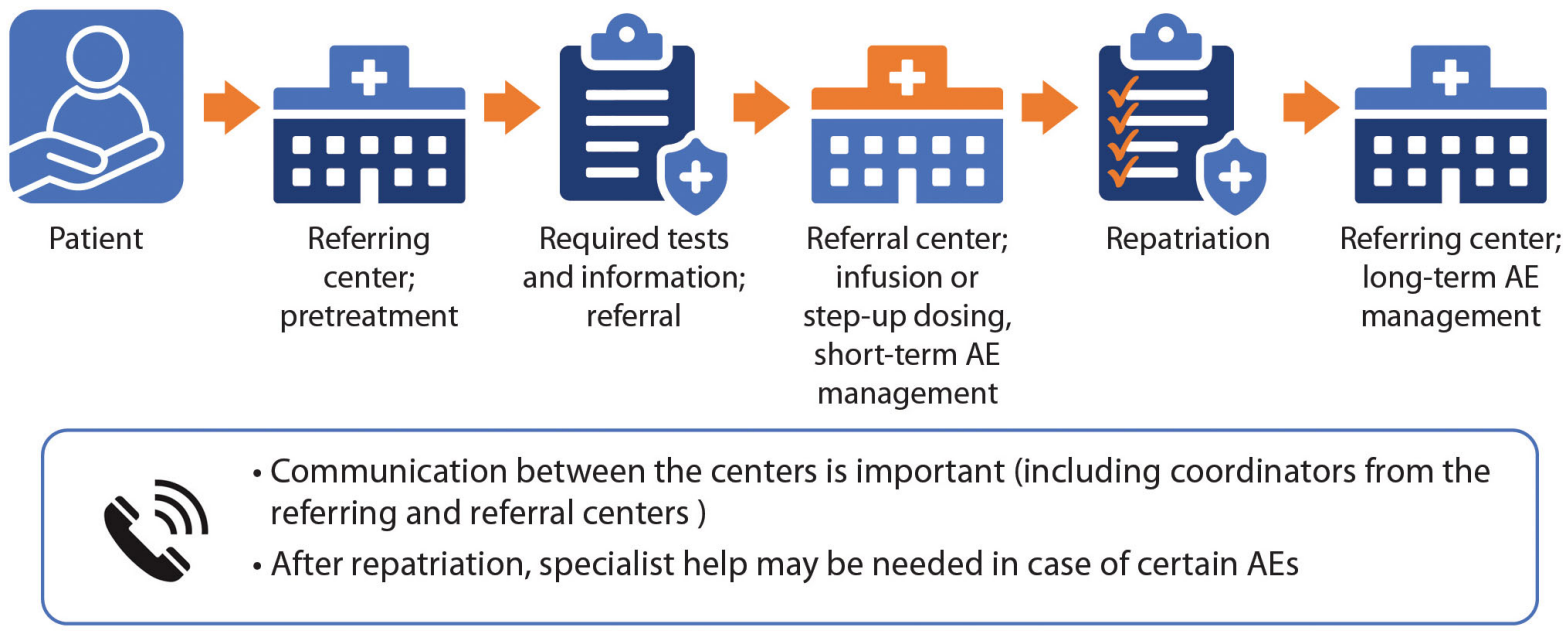
TCR referral pathway. The referring centre (local or community health centre) manages the pretreatment phase, performing required tests and sending needed information (including a detailed medical history) to the referral (tertiary) centre. The centres should communicate and agree regarding the treatment plan. The referral centre provides the infusion (if patient receives CAR T-cells) or step-up dosing (if the patient receives bsAbs), and manages short-term AEs. After repatriation, the referring centre manages treatment and long-term AEs.

The effective delivery of TCR therapies, including appropriate follow-up and AE management, is a multidisciplinary effort requiring efficient coordination and use of resources. As the use of TCR therapy in Canada increases, there is a need for clear, standardized documentation to facilitate referral processes. **Figure 1** illustrates a pathway in which patients receive care at their local clinic or community hospital (referring center) in the pretreatment phase. Some centers may be able to administer bsAbs but not CAR T-cells. Therefore, patient referral patterns for the two therapy classes may differ.

As shown in **Figure 1**, the referring center is defined as the patient’s home health care center where they have previously been treated for MM. The referral center is defined as the treatment center where they will receive TCR therapy. At present, at least 12 hospitals are equipped to deliver CAR T-cell therapy in Canada, whereas many more clinics are equipped to deliver bsAbs.

If patients are to be referred, clinicians at both centers should communicate and agree on a plan of treatment for each patient. To ensure efficient transfers of information, email communication or other communication channels should be established not only between physicians at the different centers, but also between the clerical staff and coordinators. Delays may occur if the onus is on the referring physicians to respond to information requests. Existing channels of communication for stem cell transplantation referral could be adapted for this purpose.

**Table 3** shows the key elements that should be provided by the referring center to the referral center. The referring center should organize and perform pretreatment testing. A comprehensive infection panel should be taken. Importantly, patient education, such as introductory videos, should be provided to the patient (and their care partner) before the first visit to the referral center.

**Table 3:** Key information to provide the referral center (tertiary center) when referring a patient who is eligible for T-cell–redirecting (TCR) therapy.

Essential information (“need to have”)	Additional information (“nice to have”)
**Detailed medical history**	**Imaging results (e.g., axial imaging)**
**Immunization record**	**Patient’s goals of treatment**
**Disease kinetics**	**Care partner’s goals and any additional social considerations**
**Details of performance status (ECOG score and any comorbidities that contribute to a score >0)**	**Details of any recent chronic infection**
**Previous lines of therapy, including the patient’s response to each prior LOT and timeframes during which they were administered**	
**Baseline biochemistry**	
**Complete blood count (CBC)**	
**Liver and renal function tests**	
**Echocardiogram (echo)**	
**Viral infection panel**	
**Other center-specific guidelines for pretreatment (e.g., imaging)**	

Other essential information that should be provided by the referring center includes a comprehensive medical history with the details of the patient’s disease kinetics. A summary of the patient’s responses to all previous LOT is essential to inform bridging therapy decisions. At the referral center, the patient’s preferences and goals for treatment should be discussed, and the patient and care partner(s) should be counseled regarding treatment expectations. After a treatment plan is in place, the patient should be transferred to the referral center or to the appropriate department of their community hospital to receive either a CAR T-cell infusion or step-up dosing of bsAbs. Some patients will need to travel long distances and will need lodging near the referral center. The Canadian Cancer Society offers transportation support and funding for patients undergoing cancer treatment, as well as extended travel in some areas (95). Other sources of funding, such as government programs, are also available to assist patients with travel and lodging.

#### CAR T-cell therapy initiation

Clinicians must be aware that if CAR T-cell therapy is being considered, early referral at the first sign of relapse is essential. There may be significant delays for pretreatment testing (which should be managed by the referring center), arranging lodging, travel time, and insurance approval, as well as leukapheresis and CAR-T slot assignment. For example, Ontario’s CAR T-cell therapy program requires at least 5 business days to review applications, whereas Saskatchewan’s program specifies 1-4 weeks for pretreatment testing and 4-5 weeks from cell collection to hospital admission (96, 97). In the USA, wait times of several months for apheresis slots have been reported (98), and the median manufacturing times for CAR T-cell products (between apheresis and infusion) may be up to 47 days (50, 51). During this time, bridging therapy may be required for many patients [e.g., 75% of patients in CARTITUDE-1 (10); 88% of patients in KarMMa (11)], although effective bridging therapy options may be very limited (10, 99).

Lymphodepleting chemotherapy prior to CAR T-cell infusions is performed on an outpatient basis. A CAR T-cell infusion may be followed by hospitalization, at the physician’s discretion, then repatriation to the referring center after 30 days if no infection or neurotoxicity is detected. The referring center should receive a patient transfer package and discharge report with a summary of the treatment plan, imaging results, laboratory results, recommendations for infection prophylaxis and vaccines, and contact information for the referral center. Before repatriation, the patient and care partner(s) must be educated regarding the symptoms of CRS, neurotoxicity, cytopenias, and infection. They should be provided with take-home prescriptions and instructions for contacting their treatment team if symptoms develop (including after regular business hours). Wallet cards with information about the patient’s treatment, dose schedule, signs and symptoms of CRS and neurotoxicity, and contact information for the patient’s oncology treatment team have been used to facilitate treatment for treatment-emergent adverse events (TEAEs) in case the patient presents to the emergency department, or to a new healthcare provider, with symptoms related to TCR therapy. We recommend using wallet cards, which are included in the risk management plans (RMPs) submitted to Health Canada for TCR therapies, for all patients receiving CAR T-cell therapy (100).

#### BsAb initiation

Step-up dosing of bsAbs consists of at least three doses administered over a period of up to 2 weeks (two step-up doses plus the first full treatment dose; for talquetamab, up to three step-up doses) (8, 77, 78). Pretreatment medications, including a corticosteroid, an antihistamine, and an antipyretic, should be administered before each step-up dose and before the first treatment dose (77). As discussed below, some centers may also choose to give tocilizumab prophylaxis to further mitigate the risk of CRS (101–104). Step-up dosing with pretreatment medication should be repeated if there is a delay in the treatment schedule of **≥**28 days between full doses (teclistamab) or a delay of >12 weeks between full doses (elranatamab) (77, 78). If there is a delay of >7 days after the first step-up dose of teclistamab, or a delay of >28 days after the second step-up dose of teclistamab, the step-up dosing schedule must be restarted (77). If there is a delay of >14 days after the first step-up dose of elranatamab, or a delay of >28 days after the second step-up dose of elranatamab, the step-up dosing schedule must be restarted (78). Additional guidelines regarding dose delays are given in the product monographs of these medications (77, 78). Hospitalization for 48 h after each dose should be at the physician’s discretion. To ensure effective AE management, the patient should remain close to the hospital at which step-up dosing was performed. After the first treatment cycle, the patient may be repatriated to the referring center unless infection is detected; centers should be aware that patients may need IVIg or SCIg to maintain IgG levels >400 mg/dL as prophylaxis. As with CAR T-cell therapy, wallet cards with information about the patient’s treatment, dose schedule, signs and symptoms of CRS and neurotoxicity, and contact information for the patient’s oncology treatment team are recommended (100).

### Adverse event monitoring and management

As communicated by Myeloma Canada, patients have expressed concern over the AE profiles associated with TCR therapies and their potential impact on quality of life. TCR therapies have unique AE profiles associated with their mechanisms of action and effects on the immune system (**Table 4**) (37, 105). Therefore, appropriate monitoring and effective management of TEAEs are crucial to optimize each patient’s quality of life and maximize treatment efficacy (1).

**Table 4:** Adverse events observed with TCR therapies.

Agent	Trial	All AEs, % (any grade, grade ≥3)	CRS, % (any grade, grade ≥3)	Neurotoxicity, % (any grade, grade ≥3)	Infections, % (any grade, grade 3-4)	Cytopenias, % (any grade, grade 3-4)	Skin toxicities, % (any grade, grade ≥3)	Rash-related toxicities, % (any grade, grade ≥3)	Nail toxicities, % (any grade, grade ≥3)	Dysgeusia, % (any grade, grade ≥3)	Weight loss, % (any grade, grade ≥3)	Treatment-related deaths (n)
CAR T-cell therapies												
**Cilta-cabtagene autoleucel**	CARTITUDE-1 (10, 72)	100, 100	95, 5	22, 12	58, 20	Neutropenia: 96, 95 Anemia: 81, 68 Leukopenia: 62, 61 Thrombocytopenia: 79, 60 Lymphopenia: 54, 51	none reported					6
**Ide-cabtagene vicleucel**	KarMMa (11, 165)	100, 99	84, 5	18, 3	69, 22	Neutropenia: 91, 89 Anemia: 70, 60 Thrombocytopenia: 63, 52 Lymphopenia: 27, 27 Leukopenia: 42, 39	none reported					4
Bispecific antibodies												
**Elranata-mab**	MagnetisMM-3 cohort A (no prior BCMA) (9, 146)	100, 70.7	57.7, 0	ICANS: 3.4, 0	69.9, 40.7	Neutropenia: 48.8, 48.8 Anemia: 48.8, 37.4 Thrombocytopenia: 31.7, 23.6 Lymphopenia: 26.8, 25.2 Leukopenia: 15.4, 13.0	none reported					4
**Talqueta-mab**	MonumenTAL-1; QW (8, 81)	100, 87	77, 3	10.0, 0	58, 22	Neutropenia: 67, 60 Anemia: 60, 30 Thrombocytopenia: 37, 23 Lymphopenia: 40, 40 Leukopenia: 40, 30	67, 0	47, 0	57, 0	63, 0	30, 0	None
	MonumenTAL-1; Q2W (8, 81)	100, 86	80, 0	4.5, 0	65, 16	Neutropenia: 36, 32 Anemia: 43, 23 Thrombocytopenia: 23, 11 Lymphopenia: 39, 39 Leukopenia: 18, 14	70, 2	30, 16	27, 2	57, 0	32, 2	None
**Teclistamab**	MajesTEC-1 (7, 80)	100, 94.5	72.1, 0.6 No grade 4-5 events	14.5, 0.6 ICANS: 3.0 (any-grade)	78, 52	Neutropenia: 72, 65 Anemia: 54, 38 Thrombocytopenia: 42, 22 Lymphopenia: 35, 33	none reported			1.2	none reported	6

Data from the most recent publications reporting in-depth descriptions of treatment-emergent adverse events (TEAEs) for each agent (at the time of writing) are reported. ^*^AEs, adverse events; CRS, cytokine release syndrome; ICANS, immune effector cell-associated neurotoxicity syndrome; QW, every week; Q2W, every two weeks.

**Figure 2:**
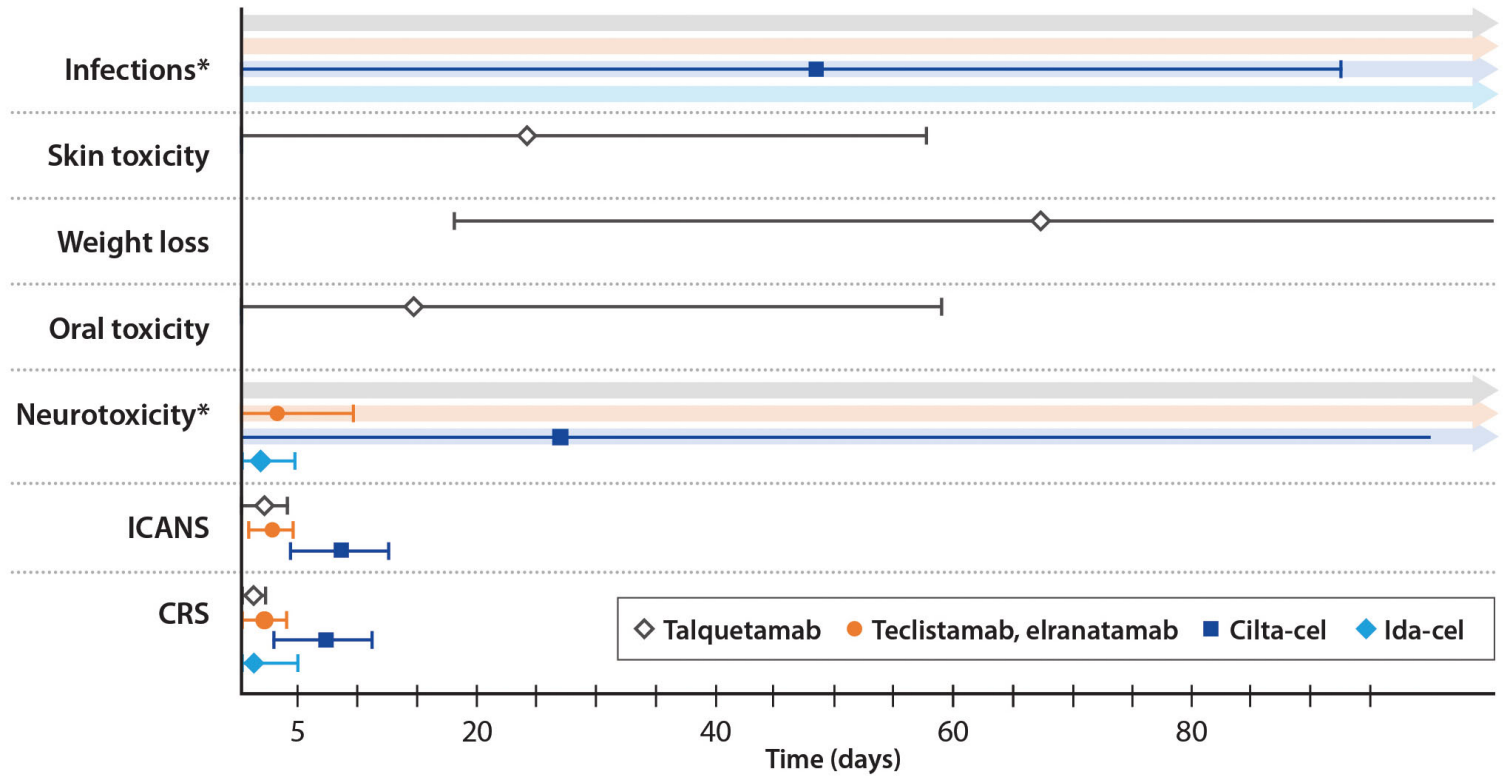
Timing of adverse events by therapy. Median values are shown; error bars indicate +/- the median duration of the event. *Neurotoxicity and infections have both been reported following the use of talquetamab and teclistamab/elranatamab, however there is no specific time window post-treatment during which these adverse events occur.

**Figure 3:**
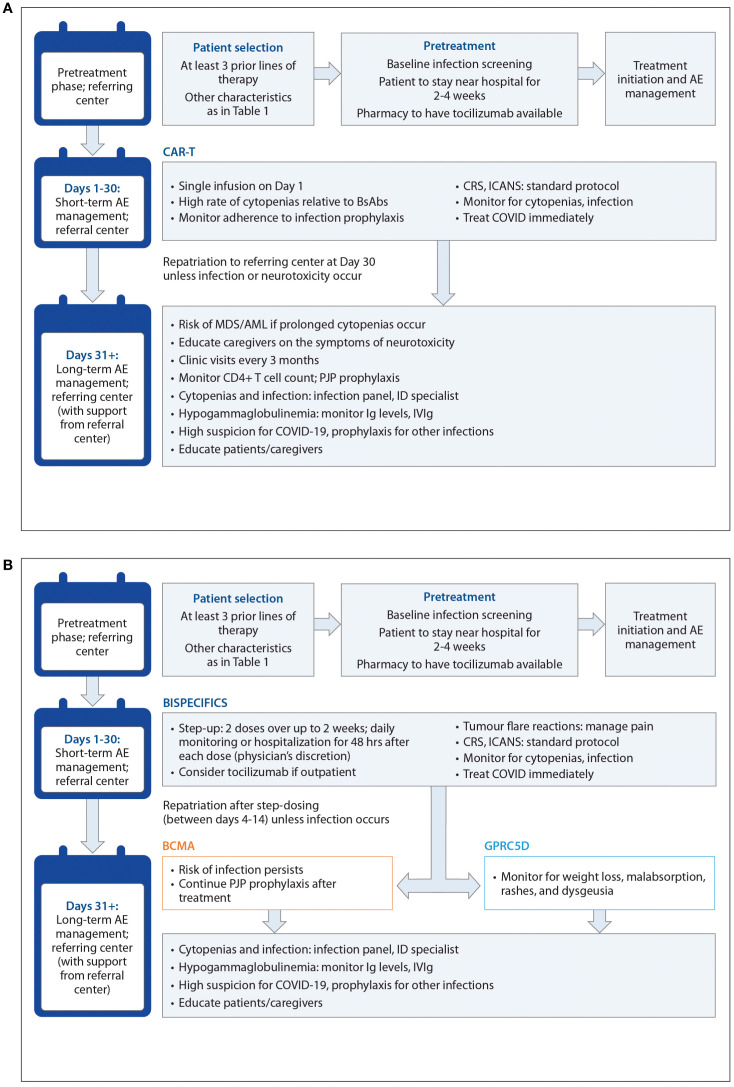
**(A)** Algorithmic approach for CART treatment and AE management The 'referring centre' is the local or community healthcare centre where the patient has been receiving treatment. The 'referral centre' is the tertiary centre administering TCR therapy. **(B)** Algorithmic approach for BsAb treatment and AE management The 'referring centre' is the local or community healthcare centre where the patient has been receiving treatment. The 'referral centre' is the tertiary centre administering TCR therapy.

CRS, neurotoxicity, cytopenias, and infections are the most commonly occurring TEAEs with TCR therapy, although TEAE patterns are target-dependent (7–11). The incidence, severity, and timing of AEs differs between CAR T-cells and bsAbs, as well as between individual agents (**Table 4**, **Figure 2**). For example, dysgeusia, skin-related toxicities, and nail-related toxicities are more common with talquetamab than with BCMA-directed bsAbs (7–9, 74). We define short-term or early AEs as those occurring within the first 30 days of treatment, which should be managed by the referral center (for those patients who are referred), and delayed or long-term AEs as those occurring after the first 30 days, which should be managed primarily by the referring center (with support from the referral center). In **Figure 3**, we present an algorithmic approach to AE management.

#### Short-term AE management – CAR T-cell therapy

CRS, neurotoxicity, cytopenias, and infections are perhaps the most common AEs of TCR therapy. CRS, which often occurs in the first few days of treatment, involves a release of inflammatory cytokines that leads to symptoms such as fever, hypotension, headache, rash and hypoxia (106, 107). With severe CRS, life-threatening complications such as renal or hepatic failure and disseminated intravascular coagulation may occur (7). In general, CAR T-cells are associated with high rates of CRS. For example, 95% of patients receiving cilta-cel in CARTITUDE-1 (n=97) experienced any-grade CRS, and 5% experienced CRS of grade ≥3 (10). Among patients treated with ide-cel in KarMMa (n=128), 84% experienced CRS of any grade, and 5% experienced CRS of grade ≥3 (11).

The onset of CRS is generally later with CAR T-cell therapy than with bsAb therapy, which has been ascribed to the slower kinetics of cytokine production (108). The peak of cytokine production is typically 2-7 days after a CAR T-cell infusion, but it may be delayed up to 3 weeks (25, 108). In CARTITUDE-1, the median onset of CRS was 7 days (interquartile range (IQR) 5-8 days) and the median duration was 4 days (IQR 3-6) (**Figure 2**) (10). Almost all cases (99%) resolved within 2 weeks (10). In the KarMMa trial, CRS occurred at 1-2 days after an ide-cel infusion and lasted 4-7 days (11). An overview of the timing of AEs is shown in **Figure 2**.

ICANS is a type of neurotoxicity that results from immune activation, cytokine production in the central nervous system (CNS), and disruption of the blood-brain barrier (109). Symptoms may include inattention, confusion, lethargy, weakness, headache, seizures, and cerebral edema (25). ICANS occurs slightly later than CRS. In CARTITUDE-1, the median onset of ICANS was 8 days (range 3-12) and the median duration was 4 days (range 1-12) (10). In KarMMa, the median time to any neurotoxic event was 2 days (range 1-10) and the median duration was 3 days (range 1-26) (11). A high degree of suspicion among clinicians, patients, and care partners, along with regular assessment, is essential.

We recommend following standard protocols, such as those issued by the ASCO and the NCCN in the USA, for the management of CRS and ICANS (25, 26). Grading of AEs should be performed according to the American Society of Transplantation and Cellular Therapy (ASTCT) guidelines; within this system, the increasing severity of hypotension and hypoxia define a higher grade (110). The standard of care includes supportive care for all-grade CRS and neurotoxicity, with additional interventions if the symptom response is poor (25). The availability of supportive medications (e.g., levetiracetam and tocilizumab) at the pharmacy should be confirmed before treatment begins. Tocilizumab is approved by Health Canada for the treatment of patients with CAR T-cell-induced severe or life-threatening CRS, but not for neurotoxicity (but note that tocilizumab should be used if neurotoxicity develops concurrently with CRS) (25). The current BC Cancer CRS management protocol recommends tocilizumab for grades 2-4 CRS (32), whereas tocilizumab is recommended for all CRS grades by the manufacturers of cilta-cel and ide-cel (50, 51). Treatment of mild CRS may inhibit progression to higher grades (32).

The administration of corticosteroids such as dexamethasone and methylprednisolone is recommended for moderate-to-severe CRS and neurotoxicity (grade ≥2) (25, 111). Dexamethasone is given intravenously (IV) at a dose of 10 mg every 6-12 h, and methylprednisolone IV at 500 mg every 12 h, followed by tapering (25). Vasopressors and respiratory support are recommended for severe CRS (grade ≥3) (25). There is no clinical consensus regarding the treatment of patients who do not respond to high-dose steroids. Severe hypoxia may require positive pressure ventilation, whereas severe hypotension calls for high-dose or multiple vasopressors (110). Pre-emptive mitigation with tocilizumab and corticosteroids is also being explored for CRS and ICANS; the IL-6 inhibitor siltuximab and the IL-1 inhibitor anakinra are under active investigation (however, at present the availability of siltuximab is variable) (25).

Care partners should be educated about these potential neurotoxicities, as the patient may not recognize their own symptoms. An immune effector cell-associated encephalopathy (ICE) score should be measured at regular intervals: while the patient is hospitalized, the measurement should be taken at least twice daily and as needed (110). After discharge, the measurement frequency may be gradually reduced. Since early recognition and timely intervention are crucial, clinicians should be aware that subtle symptoms of neurotoxicity may appear before the ICE score decreases (112). To improve accuracy, patients and care partners should be instructed not to practice the ICE test seeking to improve their scores.

Tocilizumab is not recommended for ICANS because it may worsen neurotoxicity; instead, supportive care and dexamethasone should be provided. Dexamethasone is the steroid of choice for ICANS because of its superior CNS penetration, except for grade 4 ICANS, for which high-dose methylprednisolone is recommended (25, 113). However, tocilizumab should still be used for ICANS if there is concurrent CRS. Levetiracetam may be given for seizure prophylaxis (25).

Patients receiving CAR T-cells may experience tumor flare, also known as pseudoprogression, although this is better documented in lymphoma than in MM (114, 115). Tumor flare describes an increase in tumor size that results from infiltration of immune effector cells and that is followed by clinical benefit (115, 116). Tumor flare is associated with fever, pain, leukocytosis, rash, and splenomegaly (117, 118). We recommend pain control, and if symptoms persist, steroid treatment.

#### Long-term AE management – CAR T-cell therapy

Neurotoxicities other than ICANS have been reported with anti-BCMA CAR T-cell therapy (119). These include movement disorders such ataxia, impaired hand-eye coordination, and parkinsonism; cognitive impairments such as amnesia, confusion, and mental status changes; and personality changes such as flat affect and reduced facial expression (119). These neurotoxicities occurred with a median onset of 26.5 days after an anti-BCMA CAR T-cell infusion and persisted longer than ICANS, resolving after a median 70 days (range 2-159 days) (**Figure 2**) (119). Cranial neuropathies (CNP) are also relatively common. For example, in the CARTITUDE series of clinical trials, 6.3% of patients developed CNP, with a median time to onset of 22 days; most cases were grade 2 (120). About half of the affected patients had other concurrent neurologic symptoms, such as headache. Most cases were treated with corticosteroids for a median duration of 13 days, and resolved within a median of 66 days (120).

Family members must be educated on how to recognize symptoms such as ‘brain fog’ and memory deficits. Neurological symptoms can be subtle, and patients and family members should be encouraged to report any unusual symptoms to their clinicians. If no clear explanation is found, the possibility of CAR T-related neurotoxicity should be included in the differential, and a neurology consult should be considered.

The combination of hypogammaglobulinemia, generalized immune dysfunction, and cytopenias can significantly compromise the immune responses of patients with RRMM (121). Profound hypogammaglobulinemia is an on-target effect of CAR T-cell therapy. In a Chinese multicenter study (n=40), B cell counts remained depressed until 2 months after a CAR T-cell infusion, and serum IgG levels did not recover until over a year after infusion (122). Along the same lines, a retrospective study of 82 patients treated at Mount Sinai Medical Center showed that in one-third of patients, B cell counts had not recovered to typical levels at 2 years post-CAR T (123). Thus, patients are susceptible to infections for a long period of time. The availability of IV or SC immunoglobulin (IVIg/SCIg) at the blood bank should be confirmed before treatment begins. IVIg or SCIg should be given if Ig levels drop below 4 g/L; the latter requires patients to be trained on self-injection.

Cytopenias and infections are frequent occurrences in the short and long term (**Table 4**, **Figure 2**) (25). Early cytopenias, which are thought to be caused by bridging therapy or lymphodepletion, occur within 3-4 weeks of an infusion (124). Cytopenias are the most common grade 3+ AE after CAR T-cell therapy (121). For example, among patients treated with cilta-cel (n=97) and ide-cel (n=128), grade 3-4 neutropenia occurred in 95% and 89% of patients, respectively (**Table 4**) (10, 11). A distinct pathophysiology with a biphasic course of recovery has been identified and termed immune effector cell-associated hematotoxicity (ICAHT) (121). Key risk factors for hematotoxicity include older age, disease burden, prior history of **≥**1 autologous stem cell transplant (ASCT), receipt of bridging therapy, receipt of >3 prior LOT, baseline inflammation, and previous severe CRS (121, 125). A recent study of the etiology of cytopenias indicated that systemic inflammation and low bone marrow reserves resulting from aging and/or previous therapies are linked to delayed recovery from grade 3-4 myeloid cytopenias (126). Severe ICAHT has also been linked to an increased rate of severe infection and non-relapse mortality (NRM) (127).

Granulocyte colony-stimulating factor (G-CSF) may be given to manage neutropenia, but is generally avoided during CRS events (128). Transfusions may be required for anemia and thrombocytopenia (121). If chronic thrombocytopenia develops, HLA typing for platelet transfusions should be performed, and thrombopoietin receptor agonists may be employed (although they are currently not funded for this indication in Canada) (121).

Secondary malignancy is another AE that is associated with CAR T-cell therapy, occurring in 4-16% of patients; however, prior therapy also contributes to this risk (129). In CARTITUDE -1, cases of basal cell carcinoma, myelodysplastic syndrome (MDS), lymphoma, and prostate cancer occurred (130). In KarMMA-3, an open-label, phase 3 trial that compared ide-cel with standard therapies, second malignancies were observed in both the treatment arm and standard therapy arm (131). Second primary malignancies constituted 4.3% of the AEs reported to FAERS, of which the most common were MDS, acute myeloid leukemia (AML), and dermatologic malignancies (132). T-cell lymphoma and lymphocytosis occurring within two years of CAR T-cell infusions have also been reported (133). The data so far do not distinguish between CAR T-cell therapy and other disease-related factors as drivers of these malignancies (132). However, with these findings in mind, any prolonged (≥6 months) or refractory cytopenia should be investigated (125). A bone marrow biopsy should be performed for chronic or worsening cytopenias.

Infections and cytopenias should be managed at the referring center, allowing patients to remain close to home during potentially chronic AEs. Monitoring, prophylaxis, and management of infections, hypogammaglobulinemia, and neutropenia should be per institutional guidelines. Clinicians should be aware that significant immune dysfunction occurs among patients receiving TCR therapy (134). The reactivation of latent viral infections, such as cytomegalovirus (CMV), hepatitis B, and Epstein-Barr virus (EBV) has been reported (135). Input from the referral center may be needed for opportunistic infections, with the possible involvement of an infectious disease specialist. In a retrospective study at Mount Sinai Medical Center, infections after CAR T-cell therapy were most commonly viral and occurred most often in the first 3 months after an infusion (136).

Standard infection prophylaxis must be followed with attention to adherence; COVID-19 must be identified and treated early. Clinicians should have a wide differential diagnosis for infection, and if symptoms develop, a panel that includes both common and rare pathogens should be performed. Patients must be aware of the need to investigate symptoms immediately rather than waiting to see whether the infection progresses.

We also recommend that the referring center inform the referral center of any significant or unusual symptoms, because reporting will help to build a knowledge base of TEAEs. Patients should be seen at the referring center once a month for the first 6 months after a CAR T-cell infusion. If patients are stable at 6 months post-CAR T, they should be seen every 3 months for monitoring.

Prophylaxis against bacterial and viral infections is necessary, but the duration of prophylaxis post-CAR-T remains to be validated. As described in a recent international consensus statement, antiviral prophylaxis such as acyclovir or valacyclovir should be used to prevent herpes simplex and varicella zoster (VZV) infections (137). Patients with prolonged neutropenia or a history of recurrent bacterial infections should receive bacterial prophylaxis, e.g., quinolones such as levofloxacin or moxifloxacin. To date, no standard approach to antifungal prophylaxis has been established, but we recommend that patients with absolute neutrophil counts (ANC) <0.5 x 10^9^ cells/L during the initial period after CAR T-cell therapy receive antifungal prophylaxis. *Pneumocystis jirovecii* pneumonia (PJP) prophylaxis with trimethoprim-sulfamethoxazole is essential (137). The CD4^+^ T-cell count should be monitored, and PJP prophylaxis should be continued until the CD4^+^ count is **≥**200 cells/mm^3^. Immunizations should be repeated after CAR T-cell therapy. Predictors of responses and the optimal timing of immunization remain to be determined. In particular, immunization against COVID-19 and against pneumococci (conjugate vaccine) should be repeated 3-6 months after CAR T-cell therapy (138). COVID-19 booster doses are recommended to optimize response (139). Seasonal influenza vaccination is also recommended (138). A suggested vaccine schedule, extending from 6 to 18 months after a CAR T-cell infusion, has been proposed (139).

#### Short-term AE management – BsAbs

Compared with CAR T-cells, CRS with bsAbs is less prevalent, less severe and occurs earlier (**Figure 2**) (108). The peak of cytokine release occurs as early as a few hours after infusion of a bsAb, whereas after a CAR T-cell infusion, the peak usually occurs after a few days (108). Subcutaneous administration, which is available with elranatamab, talquetamab, and teclistamab, has been associated with a delayed onset of CRS relative to IV administration (77, 78, 140, 141). In MajesTEC-1 (n=165), all-grade CRS occurred in 72% of patients, grade 3 CRS in 0.6%, and there were no grade 4 -5 events (142). In MagnetisMM-3 cohort A (n=123), elranatamab treatment led to all-grade CRS in 57.7% of patients, with no grade ≥3 events; one case of grade 3 CRS occurred in a pooled analysis of 86 patients, including 64 patients from cohort B with prior exposure to BCMA-directed therapy (9, 143). In MonumenTAL-1 (n=30, n=44), talquetamab Q2W 0.8 mg was associated with all-grade CRS in 80% of patients, with no grade ≥3 events; the QW 0.4 mg regimen was associated with all-grade CRS in 77% of patients and grade 3-4 CRS in 3% of patients (8). The median time to the onset of CRS was 2 days and the median duration of CRS was 2 days, for both talquetamab regimens (8). Teclistamab and elranatamab were both associated with a median time to CRS onset of 2 days and a median CRS duration of 2 days (7, 9).

Management of CRS involves supportive care with antipyretics, steroids, tocilizumab (8 mg/kg IV, not to exceed 800 mg/dose), IV hydration, and low-flow oxygen (25, 111). In MajesTEC-1, the use of tocilizumab at the first CRS event reduced the incidence of recurrent CRS (106).

Step-up dosing is a key component of CRS mitigation. Daily monitoring with blood work [e.g., routine hematology and blood chemistry, C-reactive protein, and ferritin (144)] should be performed during the step-up dosing. In the pivotal trials of bsAbs, almost all CRS events occurred during the step-up doses and the first treatment dose (8, 9, 80). Therefore, it is important that the patient remain in proximity to the hospital during step-up dosing. Pretreatment medications, including a corticosteroid, an antihistamine, and an antipyretic, should be administered before each step-up dose and before the first treatment dose (77). Step-up dosing with pretreatment medication should be repeated if there is a delay in the treatment schedule of **≥**28 days between full doses (teclistamab) or a delay of >12 weeks between full doses (elranatamab) (77, 78). Additional guidelines regarding dose delays are given in the product monographs of these medications (77, 78). If CRS occurs with teclistamab, premedication (but not step-up dosing) should be repeated (77). Hospitalization for 48 h after each step-up dose may be considered, at the physician’s discretion.

If the step-up dosing is performed in an outpatient setting, prophylactic tocilizumab to reduce the risk of CRS may be considered. Several studies have investigated this protocol, with early results indicating a potential benefit in reducing CRS with prophylactic tocilizumab (104). Patients in the pretreatment arm of the phase 1 GO39775 trial (n=72) received a single 8 mg/kg dose of tocilizumab intravenously 2 hours before the first step-up dose of cevostamab, a bsAb directed against FcRH5 (104). After a median follow-up of 8.5 months (pretreatment arm) and 12.8 months (no-pretreatment arm), the rate of CRS was 38.7% in the pretreatment arm and 90.9% in the no-pretreatment arm (104). Tocilizumab pretreatment did not affect response rates to cevostamab or rates of non-CRS AEs, (including infection, thrombocytopenia, and liver enzyme elevation) (104). A higher incidence of neutropenia in the pretreatment arm was noted (104). A prospective exploratory cohort of MajesTEC-1 (n =14) examined a single 8 mg/kg dose of tocilizumab administered up to 4 hours before the first step-up dose of teclistamab (101). CRS occurred in 29% of patients; all events were of grade 1-2 (101). Within the short follow-up of 1.2 months, neither the responses to teclistamab nor the incidence of grade 3/4 infection were affected by the pretreatment (101). A single-center study of 29 patients treated with teclistamab also reported no decrease in responses to teclistamab and no increase in grade 3/4 infections (145). Work carried out at Emory University Hospital examined prophylactic tocilizumab in 33 patients with triple-class refractory RRMM who were treated with teclistamab (103). In this study, tocilizumab (8 mg/kg IV; maximum dose 800 mg) was administered just before the second step-up dose of teclistamab (103). CRS occurred in 30.3% of the patients who received tocilizumab and 73.3% of the patients who did not receive it. The pretreatment cohort had lower rates of ICANS and hospital readmission, a reduced need for steroids, and fewer dose delays (103). Although the concept remains to be explored in larger studies, these early data support that prophylactic tocilizumab is effective in reducing the incidence and severity of CRS without significantly affecting responses or non-CRS AEs. This protocol could support broader adoption of outpatient step-up dosing in the future.

ICANS is less common than CRS, occurring in only 3% of patients in MajesTEC-1 and 3.4% of patients in MagnetisMM-3 (80, 146). In bsAb trials to date, most events were grade 1-2 (7–9). ICANS tends to occur later than CRS, but may be concurrent (**Figure 2**) (7).

As described above for CAR T-cells, patients receiving bsAbs may experience tumor flare, also known as pseudoprogression. Tumor flare describes an increase in tumor size that results from the infiltration of immune effector cells and that is followed by clinical benefit (115, 116). The phenomenon does not represent true disease progression (116). M-protein levels may be used to distinguish tumor flare from disease progression (147). We recommend pain control for management, and if symptoms persist, steroid treatment.

#### Long-term AE management – BsAbs

Cytopenias are frequent in patients receiving bsAbs (**Table 4**) (121). For patients who are referred for bsAb treatment rather than receiving treatment at a community hospital, infections and cytopenias should be managed at the referring center, with the input of the referral center if needed. The monitoring, prophylaxis, and management of infections, hypogammaglobulinemia, and neutropenia should be carried out by the referring center according to institutional protocols. Clinicians should be aware of the high degree of immune dysfunction observed in patients receiving TCR therapy (134). Infections caused by pathogens that are associated with T-cell depletion, such as *Pneumocystis jirovecii* and *Aspergillus* species, as well as hepatitis B and CMV reactivation, have been reported (148).

In a recent retrospective study, 41% of patients (n=37) treated with anti-BCMA bsAbs developed grade 3-5 infections, and there were two deaths due to infection (149). All responders experienced profound and persistent hypogammaglobulinemia. However, IV immunoglobulin (IVIg) was highly effective, decreasing the rate of grade 3-5 infection by 90% relative to observation (149). In contrast to CAR T-cell therapy, the cumulative risk of grade 3-5 infection during bsAb treatment persists over time, with no plateau observed (149). A viral infection panel should be performed if symptoms develop; PCR testing for pathogens is recommended. Antibody-based testing may yield false negatives because of the low B cell counts among patients with RRMM and the profound hypogammaglobulinemia induced by anti-BCMA bsAbs. COVID-19 education of patients and care partners is critical.

Prophylaxis against bacterial and viral infections should be continued as long as the patient is on bsAb treatment (irrespective of the bsAb specificity). As described in a recent international consensus statement, antiviral prophylaxis such as acyclovir or valacyclovir should be used to inhibit herpes simplex and varicella zoster (VZV) infections (137). Patients with prolonged neutropenia or a history of recurrent bacterial infections should receive bacterial prophylaxis, e.g., quinolones such as levofloxacin or moxifloxacin. All patients should receive IVIg or SCIg to maintain IgG levels >400 mg/dL. PJP prophylaxis such as trimethoprim-sulfamethoxazole (or atovaquone when an alternative is required) is mandatory and must be continued for at least 3 months after stopping treatment; the optimal duration is unknown. Annual vaccination against influenza, pneumococcal infection, and VZV is recommended, and respiratory syncytial virus (RSV) may be considered (137). National guidelines for COVID-19 vaccination should be followed, although it should be noted that vaccine responses during therapy are minimal (150). The seasonal influenza vaccine is recommended for the patient and their care partner(s) (137).

Dose modifications are being explored to reduce the risk of infections and other AEs. In a prospective cohort of MonumenTAL-1, 45 patients who responded to initial therapy switched to reduced-intensity dosing of talquetamab (0.4 mg/kg Q2W or 0.8 mg/kg Q4W), leading to a decrease in oral, dermatologic, and nail-related toxicities (151). Among patients receiving elranatamab in MagnetisMM-3, 46 responders switched to a 76 mg Q2W schedule after 6 cycles of therapy, resulting in a >10% decrease in grade 3/4 AEs while 80.4% of patients maintained or improved their responses (152). In MajesTEC-1, 104 patients who achieved a complete response were eligible to switch to Q2W dosing; 60 patients switched and 40 of these patients maintained their responses after a median follow-up of 11.1 months (153). The feasibility of this approach was supported by a retrospective study of patients who were treated with teclistamab at Memorial Sloan Kettering Cancer Center in 2022-2023 (154). In this study, 32% of patients switched to Q2W or Q4W dosing and these patients had a high six-month PFS of 94.1% (154).

Bispecific antibodies directed against GPRC5D, such as talquetamab, have unique AEs. Compared with BCMA-directed bsAbs, infection is less prevalent and the rates of some cytopenias are lower with talquetamab (**Table 4**). For example, grade 3-4 infection occurred in only 16-22% of patients who received talquetamab in MonumenTAL-1 (26% in the cohort with prior anti-BCMA therapy), but 52% and 40.7% of patients treated with teclistamab and elranatamab, respectively (80, 81, 146, 155). These differences in toxicity may be influenced by the dose intensities of each bsAb and the populations studied, as well as the biology of the target antigen; of note, a relatively low rate of grade 3/4 infections was observed with alnuctamab, an investigational BCMA-directed bsAb (156).

The period of risk for severe infections appears to be shorter with talquetamab than with BCMA-directed bsAbs: most new-onset grade 3-4 infections occur within the first 100 days of talquetamab treatment, whereas BCMA-directed bsAbs are associated with a consistent risk of severe infection throughout therapy (155, 157). At the time of writing, specific recommendations for infection prophylaxis for patients receiving GPRC5D-directed bsAbs have not been developed, and patients treated with talquetamab should receive similar infection prophylaxis to patients undergoing BCMA-directed bsAb therapy. Future recommendations may discuss bsAb target specificity in more detail (137).

Weight loss was noted in up to 32% of patients receiving talquetamab, and dysgeusia, reduced appetite, and dysphagia were also observed (8). A nutritionist consult is recommended if symptoms of weight loss are detected, and a referral to gastroenterology is recommended for malabsorption, including iron-deficiency anemia (IDA). In a phase 1 trial, dysgeusia occurred in 53.8% of patients; the talquetamab dose was reduced or interrupted in a few cases (158). According to a recent study, dysgeusia is an underrecognized toxicity in patients with MM and has also been observed in patients receiving anti-BCMA bsAbs; the mechanism is unclear (159). As yet, there is no accepted treatment for this toxicity, though improvement has been reported after breaks in therapy (151). We recommend informing patients about nonpharmacological management strategies.

Skin toxicities, rash-related toxicities, and nail abnormalities were also observed in patients receiving talquetamab (8). Although full management guidelines have yet to be developed, early experiences may guide AE management (158). Rashes should be managed with topical steroids first, followed by oral steroids if necessary. A dermatology consult is recommended if there is no response to steroids or if complications occur (157). The use of lower doses and lower dose frequencies may improve oral and skin toxicities (151).

## Discussion

The recommendations proposed here are designed to support Canadian clinicians as they expand their capabilities and develop their processes for prescribing, referring and administering TCR therapy. These recommendations coincide with a publication by the International Myeloma Working Group (IMWG) Immunotherapy Committee, with consensus recommendations for patient selection and management during treatment, baseline assessments, and TEAE risk mitigation strategies for CAR T therapy (160). As such, we view these recommendations as a flexible and evolving document that is likely to change in the future as new data emerge. Annual updates may be required, along with a keen focus on updating strategies for AE management. In particular, reduced-intensity bsAb dosing schedules are being explored, with the results so far indicating that reduced dosing and/or frequency may be employed to mitigate TEAEs (151).

CAR T-cell therapy is highly individualized and requires specialized techniques for manufacturing and delivery, whereas bsAbs are “off-the-shelf” therapies. In Canada, at least a dozen major hospitals are currently capable of delivering CAR T-cell therapies, whereas many community hospitals are equipped to administer bsAbs to complement the academic centers with greater experience. However, the optimal processes for referring patients, coordinating care, and ensuring safe and effective treatment have not been well studied (49). To expand the use of CAR T-cell therapy in Canada, hospitals will need to develop new infrastructure and provide specialized education for health care professionals (49).

TCR therapies require significant health care resources for patient referral, repatriation, and AE management. Improving the efficiency of these steps will increase access to TCR therapy and reduce the burden on the health care system. As TCR therapy advances, a move toward outpatient administration of CAR T-cell therapy and bsAb step-up dosing, supported by effective AE management, could further improve health care resource utilization and facilitate access (161). Exploratory concepts that could support outpatient administration include the use of wearable devices to monitor signs such as temperature, respiratory rate, and oxygen saturation. Wearables were found to detect initial CRS events up to 3 hours earlier than body temperature measurements alone (162).

We envision that the recommendations presented here will also enable clinicians to effectively integrate TCR therapies into their practices and to better manage specific patient populations. The proposed patient eligibility criteria are based on randomized clinical trials as well as clinical experience. We have recommended that the patient’s support network and access to a tertiary center be considered, along with an assessment of comorbidities and organ function. Patient populations in the “real world” are significantly different from clinical trial populations, often including older patients and patients with worse performance status and additional concomitant comorbidities. As more real-world data become available and as TCR agents move to earlier LOT (163), safety profiles and eligibility guidelines may change.

CAR T-cell therapy has attracted significant attention among the public, and patients may be more aware of these agents than of other therapies. According to a recent real-world study, most patients with MM (70.5%) have never heard of bsAbs, and the majority do not comprehend the risks and benefits of either therapy (164). Therefore, patient and care partner education regarding all treatment options and strategies will be necessary to facilitate informed decision-making. Care partners should always be included in therapy decision-making discussions as they will increasingly be involved in the monitoring of TEAEs. Patient education should include the efficacy and toxicity of both TCR classes, as well as considerations regarding potential hospitalizations required, travel to receive specialized treatment (more applicable to CAR T-cell therapy) and restrictions of each type of therapy.

### Limitations of the consensus statement

Given that the therapeutic landscape of MM is rapidly evolving and that TCR therapy is an area of intensive research, we have presented recommendations based on the most up-to-date evidence and clinical experience at the time of writing. We expect best practices to advance as real-world experience with TCR therapies accumulates and as clinical data mature.

## Conclusions

Here, we have presented a set of recommendations for optimizing the use of T-cell–redirecting therapy in a Canadian context. This set of recommendations, which is expected to evolve in the future, includes strategies to improve and optimize referral pathways, coordination of care, patient selection, clinical decision-making, and adverse event management. Implementation of these protocols is expected to streamline health care resource utilization, expand access to T-cell–redirecting therapies, and improve patient outcomes, including safety, for patients with RRMM in Canada.
